# Novel mutation in the *TGFBI* gene in a Moroccan family with atypical corneal dystrophy: a case report

**DOI:** 10.1186/s12920-020-00861-3

**Published:** 2021-01-06

**Authors:** Yahya Benbouchta, Imane Cherkaoui Jaouad, Habiba Tazi, Hamza Elorch, Mouna Ouhenach, Abdelali Zrhidri, Khalid Sadki, Abdelaziz Sefiani, Jaber Lyahyai, Amina Berraho

**Affiliations:** 1 Department of Medical Genetics, National Institute of Health, 27, Avenue Ibn, BP 769 Agdal, 10 090 Rabat, Morocco; 2 Laboratory of Human Pathology, Faculty of Sciences, Mohammed V University, Rabat, Morocco; 3 Research Team in Genomics and Molecular Epidemiology of Genetic Diseases, Genomic Center of Human Pathologies, Medical School and Pharmacy, University Mohammed V, Rabat, Morocco; 4 Ophtalmology Department, Hôpital Des Spécialités, Rabat, Morocco

**Keywords:** Corneal dystrophy, Thiel-behnke corneal dystrophy, *TGFBI* gene, Whole-exome sequencing, Case report

## Abstract

**Background:**

Corneal dystrophies (CDs) are a heterogeneous group of bilateral, genetically determined, noninflammatory bilateral corneal diseases that are usually limited to the cornea. CD is characterized by a large variability in the age of onset, evolution and visual impact and the accumulation of insoluble deposits at different depths in the cornea. Clinical symptoms revealed bilateral multiple superficial, epithelial, and stromal anterior granular opacities in different stages of severity among three patients of this family. A total of 99 genes are involved in CDs. The aim of this study was to identify pathogenic variants causing atypical corneal dystrophy in a large Moroccan family and to describe the clinical phenotype with severely different stages of evolution.

**Case presentation:**

In this study, we report a large Moroccan family with CD. Whole-exome sequencing (WES) was performed in the three affected members who shared a phenotype of corneal dystrophy in different stages of severity. Variant validation and familial segregation were performed by Sanger sequencing in affected sisters and mothers and in two unaffected brothers. Whole-exome sequencing showed a novel heterozygous mutation (c.1772C > A; p.Ser591Tyr) in the *TGFBI* gene. Clinical examinations demonstrated bilaterally multiple superficial, epithelial and stromal anterior granular opacities in different stages of severity among three patients in this family.

**Conclusions:**

This report describes a novel mutation in the *TGFBI* gene found in three family members affected by different phenotypic aspects. This mutation is associated with Thiel-Behnke corneal dystrophy; therefore, it could be considered a novel phenotype genotype correlation, which will help in genetic counselling for this family.

## Background

Corneal dystrophies (CDs) are hereditary bilateral primary alterations of the cornea that are not related to prior inflammation or systemic diseases; they mostly appear in the late second decade of progressive evolution [1]. These alterations have no systemic manifestations, and they present with variable shaped corneal opacities that impair visual acuity to varying degrees and often result in varying degrees of damage to corneal transparency and serious visual impairment [2]. Most corneal dystrophies tend to be autosomal dominant in inheritance, with a high degree of penetrance; few forms are inherited as autosomal recessive traits [3], such as macular corneal dystrophy, which is a corneal stromal dystrophy that leads to loss of corneal transparency and decreased vision [4]. The most commonly used classification (The IC3D: International Committee for Classification of Corneal Dystrophy) divides CDs into epithelial and subepithelial dystrophy, epithelial-stromal *TGFBI* dystrophy, stromal dystrophy, and endothelial dystrophy with new genetic, clinical and pathologic information [5, 6]. Various genes have been involved in CDs. To our knowledge, 69 disease-associated variants in the transforming growth factor beta-induced gene (*TGFBI*, OMIM 601,692) have been described as being involved in different CD subtypes in patients. The IC3D classification describes TGFBI-linked dystrophies by the recognizing that they affect multiple layers rather than being confined to one corneal layer [7–14].

Several phenotypes have been described according to corneal layer alterations and the investigation of pathogenic mutations in the *TGFBI* gene, with the exception of metabolic effects. Pathogenic mutations in the *TGFBI* gene have been implicated in various phenotypes depending on the degree of damage to the corneal layer, such as Thiel-Behnke corneal dystrophy (TBCD, OMIM 602,082), Reis-Bucklers corneal dystrophy (RBCD, OMIM 608,470), Groenouw granular corneal dystrophy type I (CDGG1), lattice corneal dystrophy I and IIIA (LCD1, OMIM 122,200 and LCD3A, OMIM 608,471) and corneal dystrophy with epithelial basement membrane (EBMD, OMIM 121,820) [15, 16]. Such dystrophy is called variant LCD by IC3D conventions [5]. Here, we present a novel heterozygous mutation (c.1772C > A; p.Ser591Tyr) in the *TGFBI* gene using whole-exome sequencing within a Moroccan family with CD.

## Case presentation

**Fig. 1:**
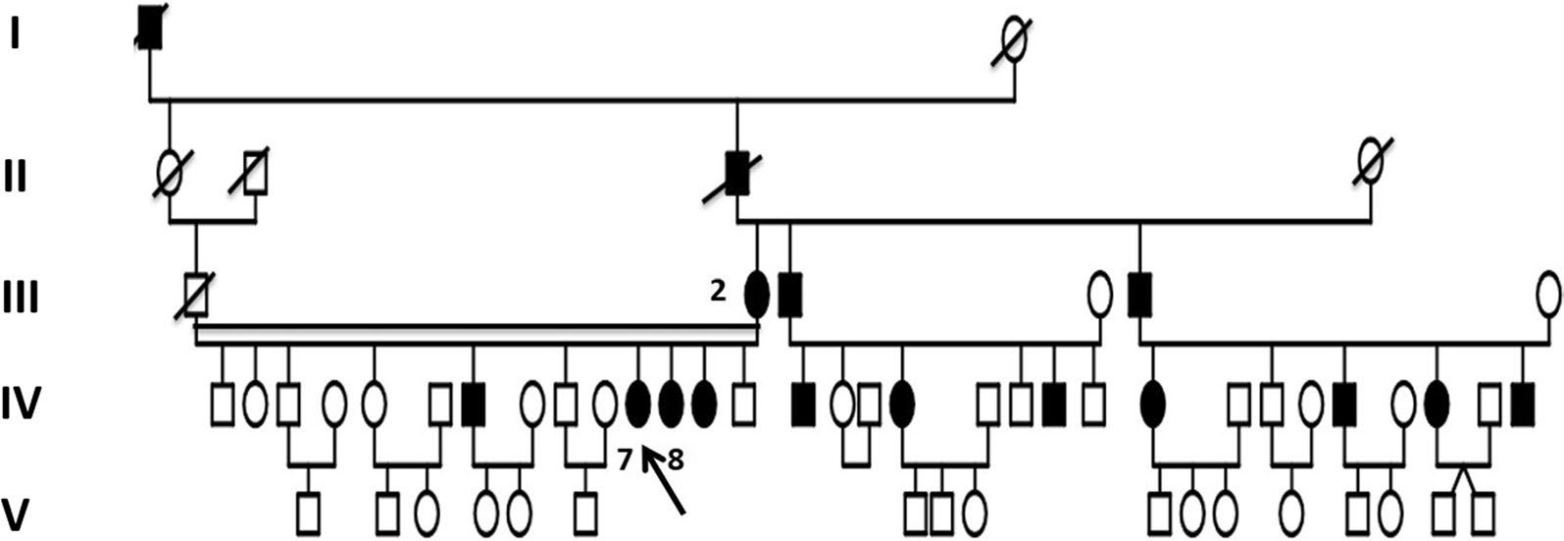
Pedigree of a four-generation family affected by Thiel-Behnke corneal dystrophy. The pedigree shows an autosomal dominant transmission of the disease. The arrow indicates the proband, the filled symbols indicate affected subjects, and open symbols represent unaffected individuals. Squares represent males, and circles represent females. A slash mark through the square or circle indicates deceased individuals

Three patients in a consanguineous family from southern Morocco were referred to our Department of Medical Genetics at the National Health in Rabat for Corneal Dystrophy. A five-generational pedigree was constructed after a thorough interview of the affected mother (III-2). The available three family members were affected (IV-7, IV-8 and III-2), including two phenotypically unaffected individuals (IV-1, IV-3). An autosomal dominant mode of inheritance was determined (Fig. 1). Clinical features of the patients are given below.

**Fig. 2:**
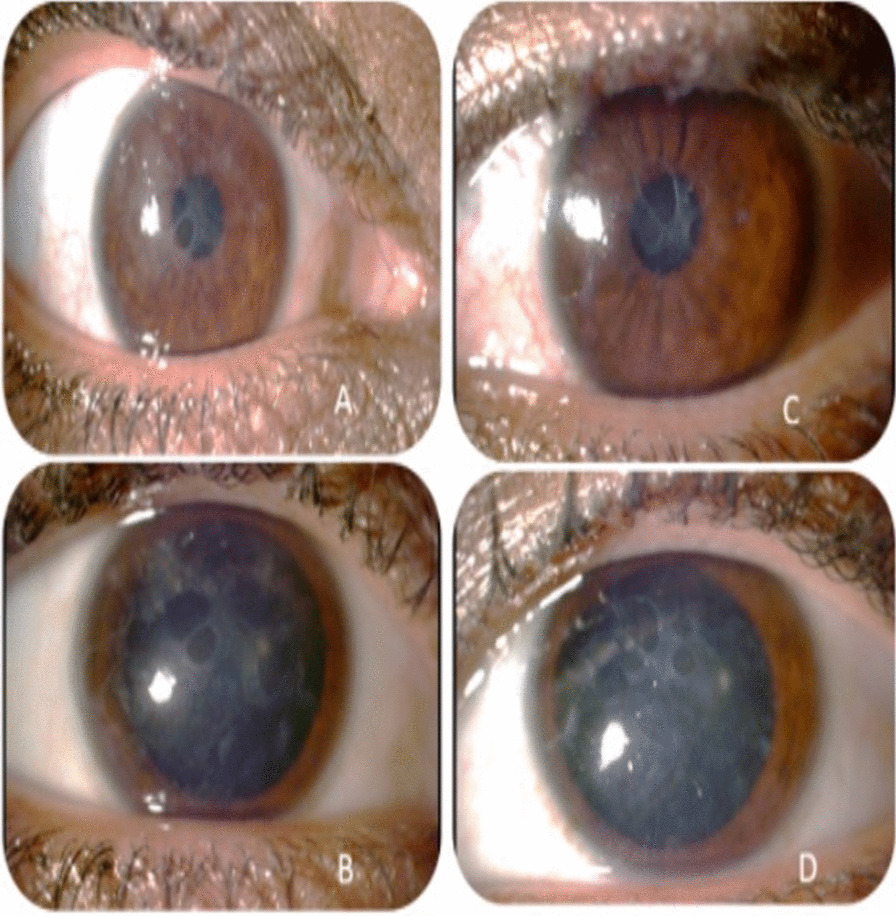
**a** Right eye without dilatations. **b** The appearance of the anterior previously poorly individualized with fuzzy edges of the right eye in the form of microvacuoles. **c** Left eye without dilation, **d** The appearance of the opacities previously poorly individualized with edges blurred left eye in the form of very visible microvacuoles after dilatation

**Fig. 3:**
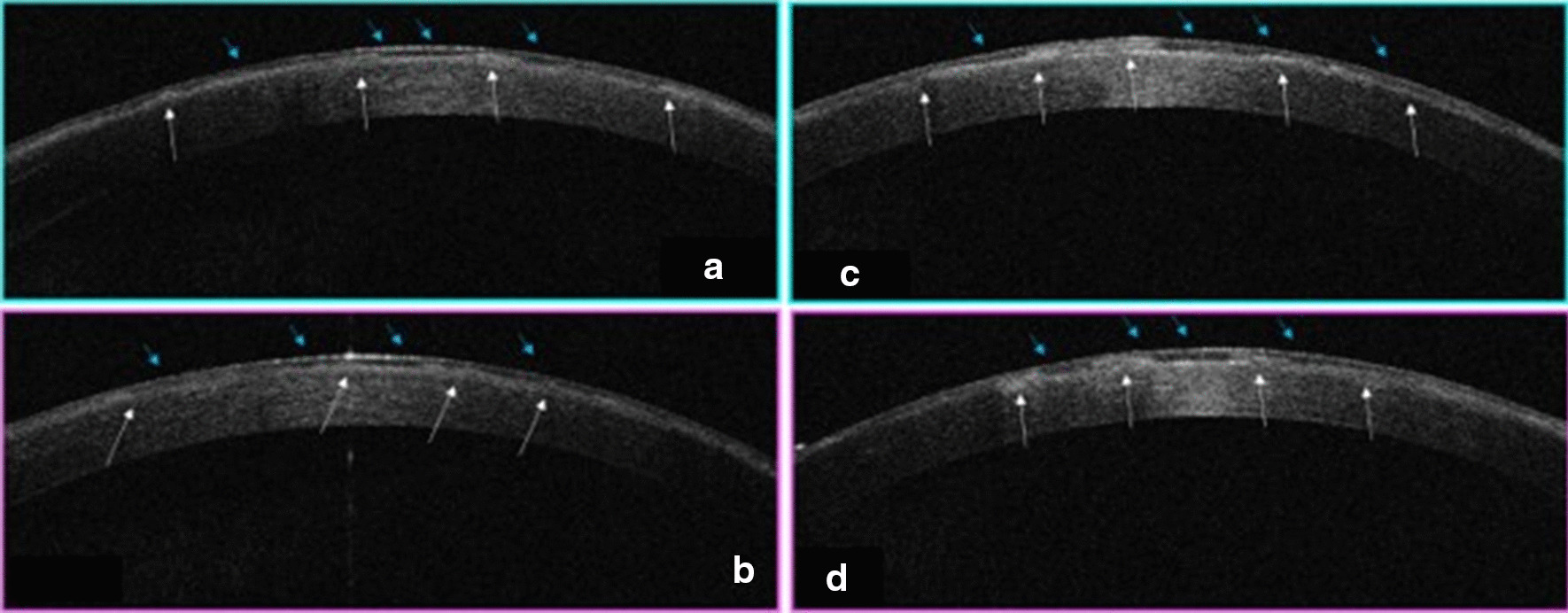
**a**, **b** Two OCT sections show the form of microvacuoles as thin patches (blue arrows) and sawtooth deposits on Bowman's membrane (white arrows). **c**, **d** The same aspect is seen in the contralateral eye

**Fig. 4:**
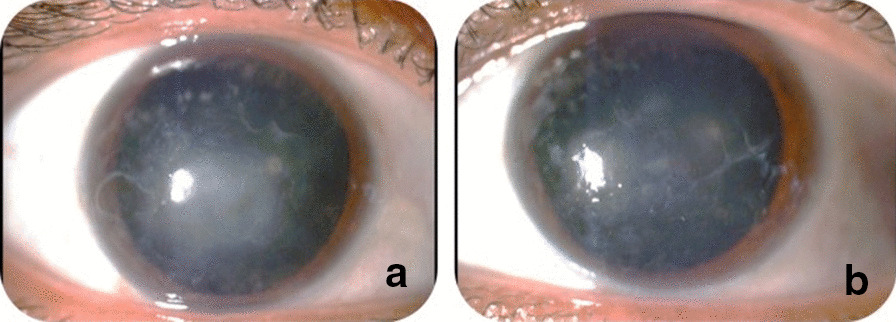
Undistinctive appearance of the anterior opacities, with blurred edges of the right (**a**) and left eye (**b**) clearly visible in the form of microvacuoles

**Fig. 5:**
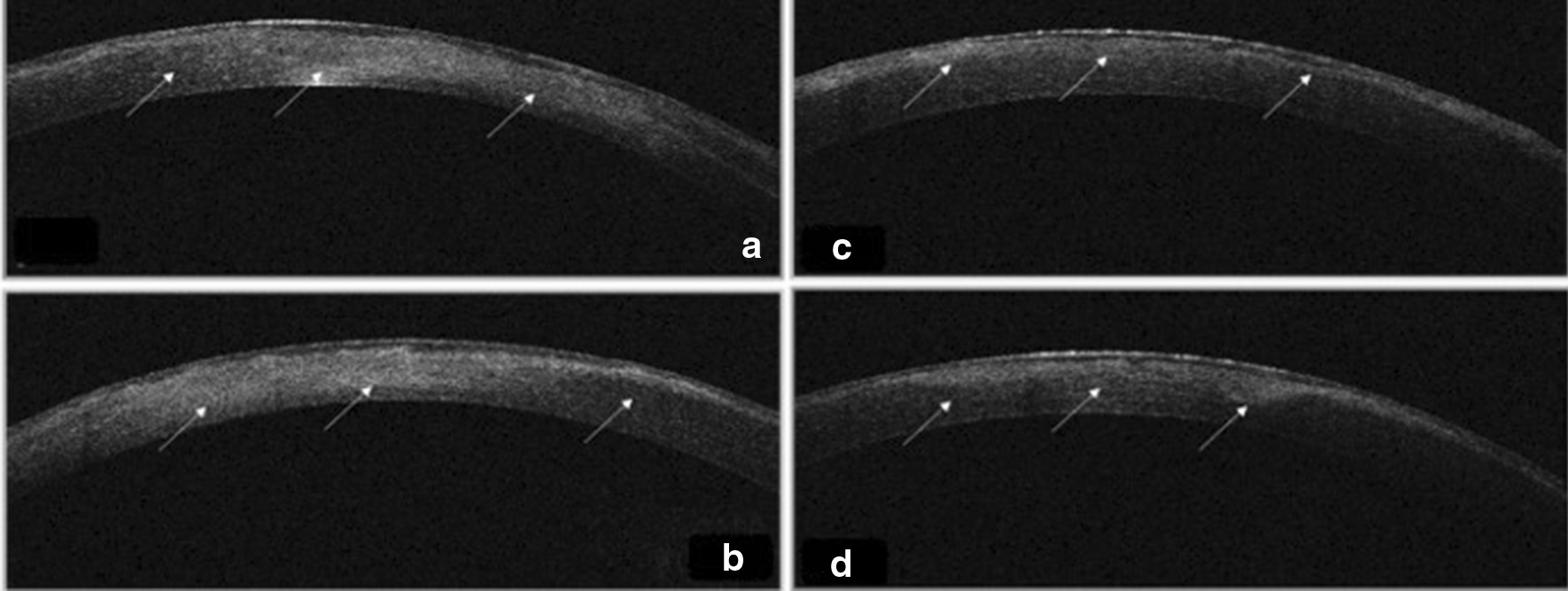
**a**, **b** Two cuts of corneal OCT of the OD. Irregularity of the anterior layers with thicker hyperreflective deposits of an apparent central opacity (arrows). **c**, **d** Two sections of OCT, irregularity of anterior epithelial and anterior stromal layers with thicker hyperreflective deposits (arrows)

**Fig. 6:**
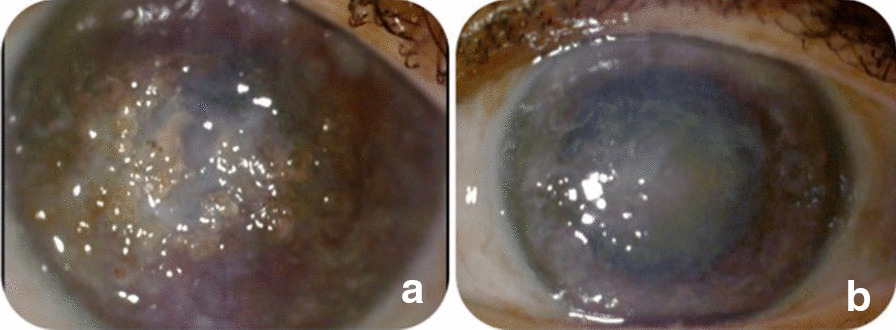
**a** The right eye has white, yellowish, gelatinous central and paracentric epithelium along with corneal opacity. **b** Central heterogeneous thickening and marks in the left eye and an obvious central opacity of blurred edges

**Fig. 7:**
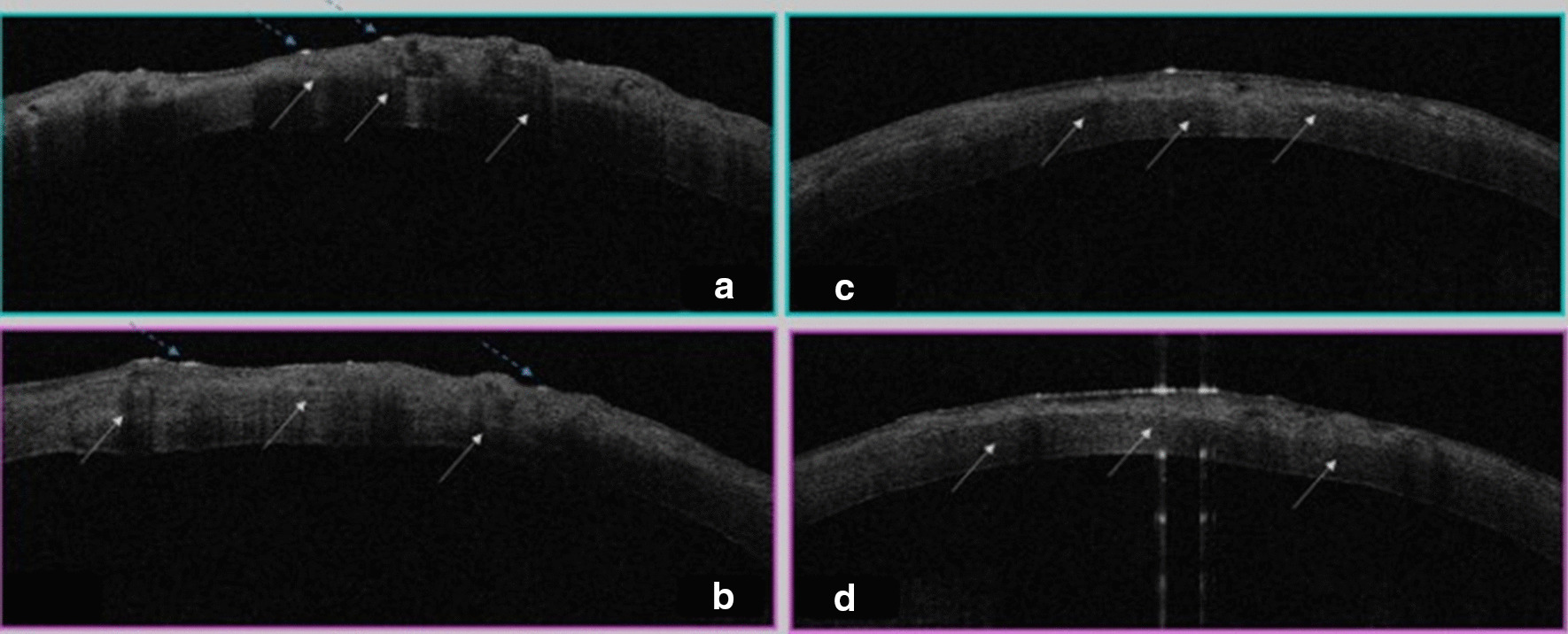
**a**, **b** Two sections of corneal OCT of OD. Deposits (white arrows); a significant reshaping of the cornea. Disorganization of the epithelial layer, absence of Bowman's membrane and blebs in the anterior intrastromal cornea (white arrows). **c**, **d**. Two sections of corneal OCT of the left eye, irregularity of the corneal surface and disorganization of the epithelial layer, absence of Bowman's membrane in different sites and the presence of a thick deposit layer of intrastromal to the centre (arrows)

- **Patient 1 (IV-7)** was a 34-year-old woman who complained of recurrent episodes of corneal pain since the age of 18 associated with a decrease in visual acuity. Her vision was 4/10 OD and 6/10 OS. Slit lamp examinations revealed anterior epithelial and stromal corneal dystrophy in the two eyes in the form of spaced microvacuoles by heterogeneous thickening of the epithelium due to thickening of an abnormal subepithelial fibrous layer and poorly individualized anterior opacities, with fuzzy edges (Fig. 2). The rest of the examination of the anterior segment was normal, including a normal iris, a clear lens, and an intraocular pressure of 17 mmHg in the ODG. The optical coherence tomography (OCT) scan showed an unevenness of the epithelial layers by a homogeneous confluent layer of hyperreflected deposits with a serrated anterior border taking the sawtooth appearance, replacing the Bowman layer and reaching the anterior stroma (Fig. 3a–d). It is thicker and becomes thinner on the periphery and disappears towards the limb. The pachymetry is 512 μm in OD and 523 μm in OS.
- **Patient 2 (IV-8)** is a 44-year-old woman who presents episodes of recurrent keratitis with a progressive decrease in visual acuity since 20 years of age. Visual acuity in OD shows that she counts the fingers at 3 m/OS at 2/10 (irremovable). Examination with the slit lamp showed anterior epithelial and stromal corneal dystrophy in the form of microvacuoles in the OD and OS groups, especially in the periphery, with heterogeneous thickening of the epithelium, which was more pronounced in the centre, giving a large central opacity with fuzzy edges in both eyes (Fig. 4a, b). She had a clear lens; ocular tone at 15 mmHg in OD 14 mmHg in OS, and a normal fundus. OCT of the cornea showed unevenness of the anterior epithelial and stromal layers with thicker hyperreflective deposits and a clear central opacity in both eyes (Fig. 5a-d), as well as pachymetry at 538 μm in OD and 543 μm in OS.
- **Patient 3 (III-2)** is a 72-year-old woman with bilateral osteoarthritis, and she reports episodes of recurrent corneal pain. For her visual acuity, in far vision OD, she could barely see the movement of the fingers/OS (she counted the fingers at 3 m). The slit lamp examination showed central and paracentric yellowish and gelatinous central and paracentric deposition in the right eye associated with a corneal opacity deeper than the previous one and affecting the epithelial layers of Bowman's membrane and the anterior stroma in the form of an epithelial fibrous layer, which covered almost the entire corneal surface (Fig. 6). The rest of the examination is hampered by the very important dystrophy of the right eye (i.e., ineclairable fundus). In the left eye, slit lamp examination showed CD involving the epithelial layers of Bowman's membrane and the anterior stroma in the form of heterogeneous thickening at the centre, associated with opacity with fuzzy boundaries and a corticonuclear cataract (Fig. 6). In the fundus, pupillary glow with flattened retina was noted. OCT of the cornea showed thin patches (blue arrows) of microvacuoles in the right eye associated with a significant loss of epithelial cells with disorganization of the epithelial layers, the absence of Bowman's membrane, and an anterior intrastromal bubble (white arrows). The pachymetry is at 586 μm (Fig. 7). In the left eye, OCT showed irregularity of the corneal surface with disorganization of the epithelial layers, discontinuous Bowman’s membrane, and anterior stromal reshaping, especially in the centre (Fig. 7). The process of corneal transplantation in this patient is ongoing.

After obtaining written consent from all participants involved in the study, we collected blood samples from family members. Blood samples were collected from five members of the family. Genomic DNA was extracted from whole peripheral blood by using the QIAamp DNA Blood Mini Kit (Qiagen Valencia, CA) strictly following the manufacturer’s protocol. Whole-exome sequencing (WES) was performed for the three patients; 500 ng of fragmented DNA was obtained by enzymatic fragmentation with the Kapa Hyper Plus Kit (KapaBiosystems Inc. Wilmington, MA, USA) and was amplified according to the manufacturer instructions and subjected to enrichment with SeqCap EZ Human Exome v3.0 Roche Nimblegen (Roche, Basel, Switzerland).

The Illumina HiSeq 2500 system was used to sequence 64 enriched megabases in fast-running double-ended mode (2 × 100 bp). bcl2fastq v1.8.4 (Illumina) was used to convert the original data (bcl file) into a fastq file. The sequence was analysed according to GATK best practice recommendations; BWA-MEM was used for mapping, and GATK (haplotype calling program) was used for variant calling. Variant Studio (Illumina) was used for annotation and filtering steps.

Candidate variants were selected using an autosomal dominant mode of inheritance according to the following criteria: i) heterozygous variants, ii) nonsynonymous variants, iii) variants predicted as pathogenic or likely pathogenic, iv) variants with a minor allele frequency (MAF) of < 0.01 were selected from the 1000 Genomes Project (http://www.1000genomes.org/) and ESP6500 exome project (https://evs.gs.washington.edu/EVS/) and v) segregation analysis. Candidate variants in favour of clinical manifestations and passing the criteria were validated by Sanger sequencing.

The established variants were cross-checked with the 1000 Genomes database (http://www.1000genomes.org/) with the Exome Variant Server (http://evs.gs.washington.edu/EVS/), HGMD (http://www.biobase-international. com/product/hgmd) and with the ClinVar database (http://www.ncbi.nlm.nih.gov/clinvar/).

To confirm the mutation detected by exome sequencing, standard PCR was carried out to amplify exon 10 of the *TGFBI* gene by using the *TGFBI*_F:5′-GACCAGGCTAATTACCATTCTTG-3′ and *TGFBI*_R:5′-TGAGATATGTCCTGGAGCCC-3′ primer pair. Amplification products were electrophoresed on a 1% agarose gel. Sanger sequencing was performed with dye terminator chemistry (ABI Prism BigDye v3.1) and run on an automated sequencer using the 3130 Genetic Analyzer (Thermo Fisher Scientific). The results obtained were aligned with the reference genome (GRCh37/hg19) and then analysed by DNA variant analysis software (Mutation Surveyor® software).

The transmission form of this family was consistent with autosomal dominant inheritance (Fig. 1). Clinical examinations demonstrated bilaterally multiple superficial, epithelial and stromal anterior granular opacities in different stages of severity among three patients of this family. The three patients with the same mutation shared a mixed phenotype with a superficial form of granular corneal dystrophy (GCD) type 1 and TBCD patterns.

**Fig. 8:**
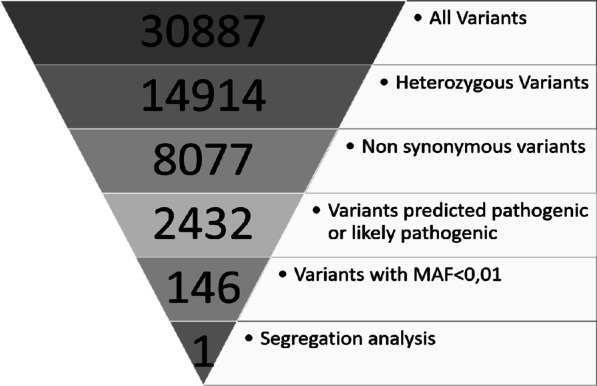
Bioinformatic analyses of whole-exome sequencing in three family members (IV-7, IV-8 and III-2)

**Fig. 9:**
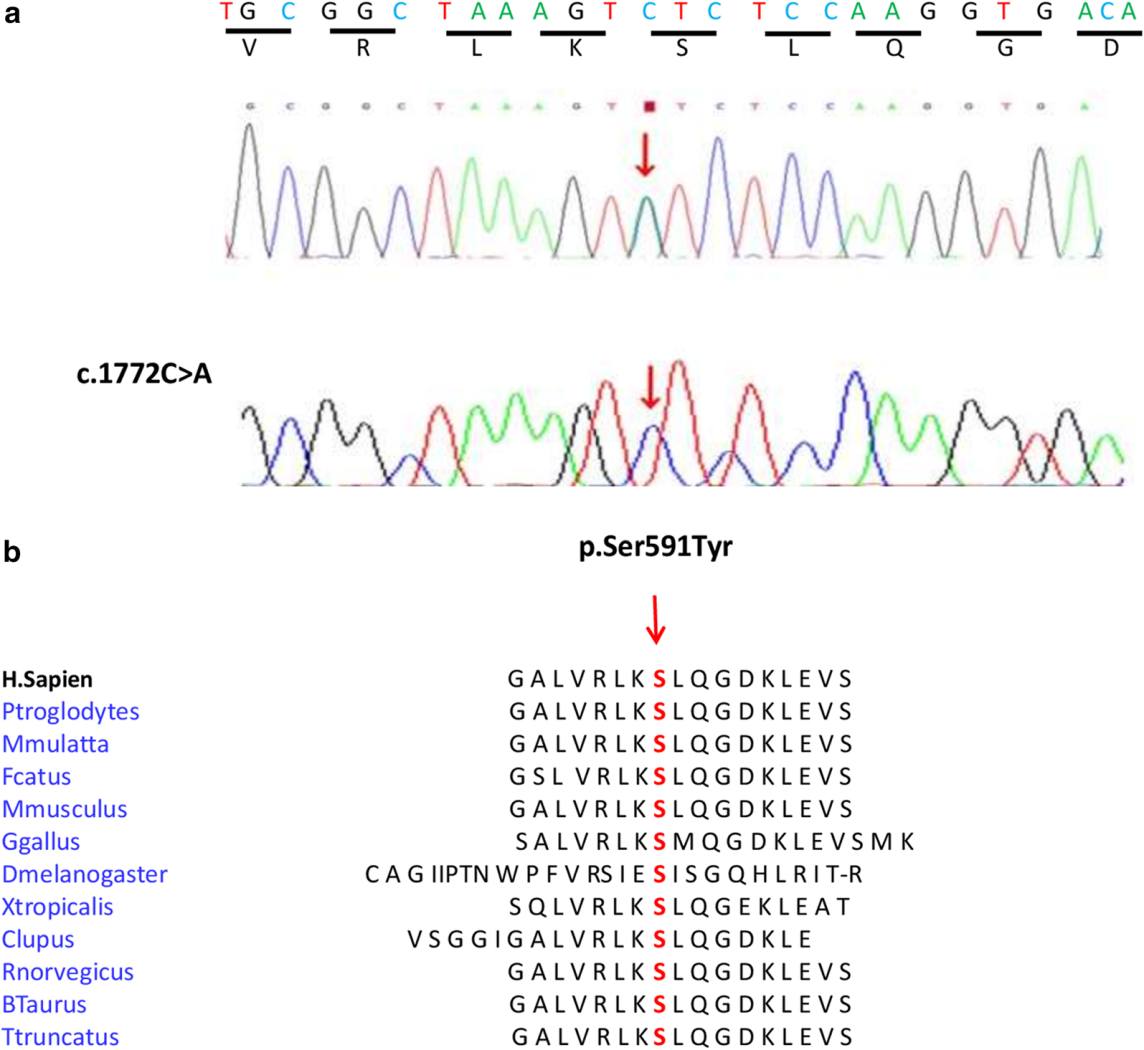
**a** Electropherograms showing the c.1772C > A heterozygous mutation in the patients (IV-7, IV-8 and III-2) and the absence of the mutation in the unaffected individual (IV-1, IV-3). Arrows indicate the region of the mutation. **b** Orthologous protein sequence alignment of *TGFBI* from different species. The mutated residue demonstrating conservation of serine (S) at codon 591 is indicated by the arrow. *TGFBI* and transforming growth factor β induced atypical corneal dystrophy. (https://www.ensembl.org/Homo_sapiens/Tools/Blast)

Whole-Exome Sequencing and Variant Validation**:** WES was performed on three family members (IV-7, IV-8 and III-2). The analysis of the data of the three patients shows 30,887 variants in 19,712 genes. After filtering, this number was reduced to a single allelic variant in the *TGFBI* gene. The filtering of variants is illustrated in Fig. 8. Only a heterozygous mutation (c.1772C > A; p.Ser591Tyr) in the *TGFBI* gene, identified in these three patients, was proposed as the potential pathogenic mutation within this family (Fig. 9a).

Bioinformatics analysis using Polyphen-2 and SIFT suggested that this mutation was probably damaging. A high degree of conservation of this amino acid was demonstrated by a score of 5.52 calculated by Genomic Evolutionary Rate Profiling (GERP) http://mendel.stanford.edu/SidowLab/downloads/gerp/index.html. Comparative amino acid sequence alignment of *TGFBI* across different species revealed that this mutation occurred at highly conserved positions (Fig. 9b) (https://www.ensembl.org/Homo_sapiens/Tools/Blast). The mutation was confirmed by Sanger sequencing in the affected sister and mother and two unaffected brothers.

Furthermore, the variant was not present in the GnomAD browser (accessed July 2020) or in our in-house WES database of 100 unrelated individuals of Moroccan ethnicity who had undergone WES for diseases other than corneal dystrophy.

The genomic and clinical data both supported a diagnosis of Thiel-Behnke corneal dystrophy in this family.

## Discussion and conclusions

Thiel-Behnke corneal dystrophy was first described in 1967 and is characterized by subepithelial honeycomb-shaped corneal opacities in the superficial cornea, progressive visual impairment and autosomal dominant inheritance [17]. Symptoms begin with recurrent corneal erosion during childhood (first and second decade of life), and visual acuity is affected later in life. TBCD can be particularly confused with RBCD during the first two decades of life.

TBCD presents as bilaterally cross-linked epithelial opacities in honeycombs with remarkable symmetry with the accumulation of reticular flecks at the level of the Bowman membrane.

The peripheral cornea is typically not involved but can be affected with time in older patients. While RBCD has a geographic-like phenotype, TBCD has a honeycomb-like phenotype. Histopathologically, TBCD is characterized by alternating irregular thickening and thinning and a focal absence of the epithelial basement membrane.

The Bowman layer is replaced by a superficial fibrocellular scar with a pathognomonic wavy sawtoothed pattern or “curly” fibres. The prevalence of TBCD is unknown. Only a few cases have been reported in European populations, the United States, Japanese populations and other ethnicities, but none have been reported in North African populations [18, 19]. To our knowledge, mutations (p.Arg555Gln; p.Arg555Trp) in the *TGFBI* gene and in the same position have also been reported by Xiang et al. and Yu Y et al. [20, 21] and were correlated with TBCD [7, 22, 23]. In this study, we identified a novel mutation in the *TGFBI* gene (c.1772C > A; p.Ser591Tyr). To our knowledge, the association of the p.Ser591Tyr mutation in TBCD has not been reported previously and is not listed in LOVD v.3.0 (Leiden open variation database). The *TGFBI* gene is located on chromosome 5q31.1 and encodes a protein of 17 exons composed of 683 amino acids [24, 25]. This protein is used for both cell adhesion and the integrin recognition sequence. However, extracellular matrix protein *(*TGFBI*p)* contains an EMILIN-like domain rich in N-terminal cysteine, four consecutive and highly homologous domains of fasciclin 1 (FAS1), and a C-terminal arginine-glycine-aspartic acid motif [26, 27]. All pathogenic variants caused the accumulation of insoluble extracellular material in the cornea [28]. The accumulation of full length and fragments of aberrant TGFBIp has been demonstrated in corneal deposits [29]. It appears that mutations are also likely to change protein degradation pathways and impact the structure and stability of aggregated proteins [24]. Most gene *TGFBI* mutations associated with CD are heterozygous, and some patients with homozygous mutations have a more severe phenotype, indicating that the potential toxic effect has a dose response effect [30]. The p.Ser591Tyr mutation is predicted to be damaging and is located in the fourth domain (domain Fasc 4). All the *TGFBI* mutations described, except p.Arg124Cys, are located exactly at the boundary or in this domain. This domain is suspected to have a specific action directly or by interaction with an unknown protein [31].

The two hotspots in the *TGFBI* gene have been reported to be associated with the most common corneal dystrophies; the p.Arg555Trp mutation in GCD1 and the p.Arg124His mutation in GCD2. The p.Arg124Cys mutation was reported in LCD1, whereas the p.Arg124Leu mutation was reported in anterior stromal CD, and the p.Arg555Gln mutation was reported in RBCD [32].

The management of CDs varies with the specific disease. For the treatment of this pathology, corneal transplantation is the recommended treatment, a major limitation being the recurrence of posttransplant corneal deposits; however, some patients are treated with drugs, and others use methods that suggest removal of the abnormal layer, such as phototherapeutic keratectomy or deep anterior lamellar keratoplasty (DALK) [27].

Regarding our family case, the chronological evolution of this new dystrophy indicates early treatment by photokeratectomy in an earlier stage with a promising postoperative result. The particularity in this family is the evolution and preservation of the posterior layer, which will lead to adapted surgery.

In summary, in this paper, we report a novel pathogenic mutation in the *TGFBI* gene that could be a novel phenotype genotype correlation. The clinical and genetic results of our study may further expand the spectrum of TGFBI-linked CDs and may be of great help in genetic counselling for this family.

## Data Availability

The datasets analyzed during the current study are available in the zenodo; number: 4288687; direct link: https://zenodo.org/record/4288687#.X70HP3BKi1t Repository. The novel variant has been submitted to the specific gene variant database Leiden Open Variation Database (LOVD; individual number: 00319928) https://databases.lovd.nl/shared/variants/0000703906) and ClinVar database (accession number: SCV001442510.1) https://www.ncbi.nlm.nih.gov/clinvar/submitters/505877. The download links of public data used in this study are listed below: ftp://ftp.ensembl.org/pub/grch37/current/fasta/homo_sapiens/dna and then analyzed by DNA variant analysis software (Mutation Surveyor® software). The established variants were cross-checked with the 1000 genomes database (http://www.1000genomes.org/), with the Exome Variant Server (http://evs.gs.washington.edu/EVS/), HGMD (http://www.biobase-international. com/product/hgmd) and with the «clinvar» database (http://www.ncbi.nlm.nih.gov/clinvar/).

## Abbreviations

CD: Corneal dystrophy
WES: Whole-exome sequencing
IC3D: The international committee for classification of corneal dystrophies
TGFBI: Transforming growth factor beta induced
TBCD: Thiel-Behnke corneal dystrophy
RBCD: Reis-Bücklers corneal dystrophies
CDGG1: Groenouw granular corneal dystrophy type I
LCD: Lattice corneal dystrophies
EBMD: Corneal dystrophy with epithelial basement membrane
GCD: Granular corneal dystrophy
OCT: Optical coherence tomography
FAS1: Fasciclin 1
DALK: Deep anterior lamellar keratoplasty
